# Lights and Shadows of Sepsis Management: Challenges and Future Perspectives

**DOI:** 10.3390/ijms24119426

**Published:** 2023-05-29

**Authors:** Alessandro Russo, Rita Pallone, Enrico Maria Trecarichi, Carlo Torti

**Affiliations:** Infectious and Tropical Disease Unit, Department of Medical and Surgical Sciences, ‘Magna Graecia’ University of Catanzaro, Viale Europa, 88100 Catanzaro, Italy; pallonerita@gmail.com (R.P.); em.trecarichi@unicz.it (E.M.T.); torti@unicz.it (C.T.)

## 1. Introduction

The complex interaction between microorganisms, the host’s immune response, and the release of pro- and anti-inflammatory factors influence the evolution of sepsis. According to the current definition, sepsis should be considered a severe, potentially fatal organ dysfunction caused by an inadequate or dysregulated host response to infection [1]. Overall, the global incidence rate is about 400 episodes per 100,000 person years, and the number of cases in industrialized countries is continuously increasing [1]. As a matter of fact, it is recognized that a prompt diagnosis and an appropriate treatment approach are the milestones of sepsis management. However, despite medical advances in recent years, the mortality rate is over 30%, being one of the leading causes of mortality and morbidity worldwide, with a greater impact in medium- and low-resource countries.

From a pathophysiological point of view, the interaction between some structural components (such as lipopolysaccharide for Gram-negative bacteria or lipotecoic acid for Gram-positive bacteria) and the production of bacterial exotoxins can cause a systemic activation of the immunological compound, also found through the expression of superantigens. Specifically, TLRs (Toll-like receptors) expressed on monocytes and macrophages recognize PAMPs (extracellular pathogen-associated molecular patterns) and DAMPs (damage-associated molecular patterns) released from damaged endogenous cells. As a result of this mechanism, massive amounts of pro-inflammatory cytokines are released, resulting in increased nitric oxide (NO) production, capillary permeability and vasodilation, and thus hypotension. Impaired coagulation, if massive, can lead to the development of disseminated intravascular coagulation with the excessive consumption of coagulation factors and platelets. Vasodilation, the extravasation of fluids, and disseminated thromboses have, as an evident consequence, hypoperfusions of organs and tissues, resulting in a possible development of multi-organ failure. 

Many studies highlight the need for a clearer understanding of the pathophysiological mechanisms underlying sepsis in order to achieve an early and timely diagnosis and to set up strategic therapies that are increasingly focused on precision medicine. The recent Special Issue “The Interplay between Host Defense, Infection and Pathogen Virulence in Sepsis and Septic Shock” highlighted important aspects of the diagnoses and management of septic patients. As reported above, the dysregulated host response to infection, leading to sepsis and septic shock, is a life-threatening event that is associated with high mortality rate, despite advances in organ support and antimicrobial therapy. Despite the implementation of international guidelines supporting early goal directed therapy, recent randomized trials have demonstrated that these interventions do not improve the survival of septic patients.

This evidence warrants an urgent clarification of the molecular mechanisms underlying clinical response in patients with sepsis or septic shock. The key to improving these processes lies in acquiring in-depth knowledge of the intricate interplay between host defense, infection, and pathogen virulence, as well as the timing and type of interventions that are most effective according to the personal characteristics of individual patients. Of importance, the pharmacokinetic (PK) and pharmacodynamic (PD) properties of antibiotics should be considered because of changes in clearance and volume of distribution that are frequently observed in critically ill patients, with the potential to influence the concentration of the drug at the sites of infection.

## 2. Different Aspects of Sepsis Management

Based on the aforementioned knowledge of the mechanisms related to the progression from sepsis to septic shock and the adequate management of patients, including choice and dosages of antimicrobials, the results are crucial to improve outcome of septic patients. Specifically, the manuscripts published in this Special Issue [2,3,4,5,6,7,8] highlighted some important points:

(1) the long-term effects of chronic neonatal inflammation in the course of neonatal sepsis and thus the need for an early diagnosis;
(2) the attempt to identify new diagnostic strategies using more sensitive and specific biomarkers aimed at achieving increasingly rapid health interventions and therapeutic improvements;
(3) the limitations of current diagnostic techniques, especially during polymicrobial sepsis;
(4) the key roles of the pathophysiological mechanisms of sepsis to identify new diagnostic and therapeutic strategies, especially for multidrug resistant (MDR) pathogens.

It should be considered that sepsis causes high rates of morbidity and mortality also in the neonatal intensive care unit (NICU). The review conducted by Maddaloni et al. [2] focused on the key role of presepsin (P-SEP) as a promising and reliable biomarker for the early diagnosis of sepsis in neonates. P-SEP, a soluble fraction of CD-14, is a protein expressed by all immune system (IS) cells, and it is secreted by the liver and monocytes. Although currently available studies need further clarification and investigation, P-SEP could be not affected by perinatal confounding inflammatory factors compared to other biomarkers used in clinical practice such as procalcitonin (PCT) and the C-reactive protein (CRP). In addition, it would appear that the P-SEP blood levels are related with the severity of sepsis, as well as being useful in monitoring response to antibiotic therapy. 

Joubert et al. [3] focused their attention on the host–pathogen interactions between premature infants and nosocomial-coagulase-negative staphylococcal infections. According to numerous studies, *Staphylococcus epidermidis*, a ubiquitous skin commensal, is reported to be the main etiology in late-onset neonatal sepsis (3–28 days after birth). It is unclear what long-term chronic neonatal inflammation might entail, but it is probable that the damage could affect cognitive pathways, impairing neuronal development. Thus, laying the groundwork for the early diagnosis of neonatal sepsis in preterm infants remains a challenge and a goal to be achieved. This is because, even today, diagnosis is based on microbial blood cultures that are burdened by numerous limitations. Medical advances and molecular and sequencing techniques are needed for the development of alternative preventive, diagnostic, and therapeutic tools to combat neonatal infections. 

As a matter of fact, the management of critically ill patients admitted to an intensive care unit (ICU) remains a challenge for physicians. The majority of patients undergo continuous renal replacement therapy (CRRT) and/or extracorporeal membrane oxygenation (ECMO) that can directly affect the response to sepsis and the efficacy of antibiotic therapies. Köhler et al. [4] remarked and implemented the available scientific knowledge about modulation of the host defense by CytoSorb. Although the indication, timing of efficacy, and duration of therapy have not yet been fully elucidated, just as a decrease in mortality during septic shock has not been clearly demonstrated, CytoSorb is currently the most widely used hemoadsorption and immunomodulation procedure that aims to eliminate pro-inflammatory substances such as cytokines by extracorporeal circulation, acting on hyperinflammation. Evidently, more studies will be needed to test CytoSorb as a routine practice in ICUs. 

The role of precision medicine was the topic addressed by Webber et al. [5]. The authors inquired about the identification of early biomarkers in the diagnosis of sepsis. Plasma inducible nitric oxide synthase (iNOS) would appear to play an important role in the induction of a cytokine storm and thus could be a candidate not only as a specific biomarker but also as a novel therapeutic target for the treatment of sepsis. This has led to the development of an anti-microvesicle monoclonal antibody associated with iNOS, with the aim of neutralizing it. However, further studies are needed to confirm and ascertain the additional value of this biomarker in clinical practice. 

Another important topic was focused on by Doualeh et al. [6], who showed that the polymicrobial etiology of sepsis was associated with worse outcomes than in monomicrobial sepsis. In last years, many efforts have been made to develop advanced molecular techniques, with the aim of overcoming the limitations of current blood culture diagnostic techniques, such as slow response times, low sensitivity, and varying growth rates that may lead to the inappropriate prescription of antibiotic therapy. In addition to host–pathogen interactions, pathogen–pathogen interactions also drive host inflammatory processes through the mechanisms of co-infection and the additive effect. Research on the development of faster and more sensitive methods for the diagnosis of sepsis is, therefore, critical. Among molecular techniques, quantitative PCR (qPCR) has been found to be among the fastest and cheapest; 16S ribosomal RNA (rRNA) gene sequencing or cell-free DNA sequencing may also be feasible due to their high accuracies. However, critical issues of molecular techniques, such as amplification errors due to target mutations in the case of nucleic acid amplification, assay inhibition, contamination, delivery time, and the inability to determine whether the sequenced or amplified DNA comes from viable or dead microorganisms, are also highlighted in the study.

The review by Krishnan et al. [7] focused on sepsis caused by carbapenemase-resistant *Acinetobacter baumannii* (CRAB), which is responsible for lethal sepsis and high mortality rates in hospital settings. The authors focused on attempting to develop new therapeutic options, such as the development of a novel peptide (R-Pro9-3D) with cytotoxic, antibacterial, and immunosuppressive effects, which act directly on bacterial LPS. Specifically, the study found that R-Pro9-3D had an inhibitory effect on NO production, which was responsible for acute and chronic hyperinflammation, and thus its inhibition appeared to counteract the release of pro-inflammatory factors and the organ damage that inevitably resulted.

Finally, the review carried out by Lazzaro et al. [8] shed significant light on the mechanisms and interactions between host defenses, infections, and clinical statuses in septic patients. Having a clear understanding of the pathophysiological mechanisms underlying sepsis, before focusing on diagnostic and therapeutic approaches, is critical to understanding the importance, challenges, and complexities of this syndrome. The mechanisms responsible for the onset of sepsis are very complex and not yet fully elucidated, but we know that bacterial components can directly stimulate the activation of an innate immune response, and multiple microbial products are recognized early by the complement system. As a matter of fact, specific receptors that lead to the activation and release of pro-inflammatory factors can stimulate cellular damage by using a cytotoxic effect. These effects are perhaps implemented by the virulence factors of MDR pathogens. 

## 3. Challenges and Future Perspectives

In recent years, an increased frequency of Gram-negative MDR pathogens such as MDR *Acinetobacter baumannii* (MDR-AB) and carbapenemase-producing *Klebsiella pneumoniae* (KPC-Kp) has been observed. It is important, therefore, to develop new diagnostic techniques to obtain a fast microbiology with early microbiological reports. These techniques can include nucleic acid amplification technologies (NAATs) that amplify nucleic acid sequences to a detectable level and identify the infecting agent or the status of the immune response. The detection of bacterial DNA fragments by a real-time polymerase chain reaction (RT-PCR) in blood samples and the detection of 16S rRNA fragments of Gram-positive and Gram-negative bacteria or 18S rRNA fragments of *Candida* spp. seem to be very promising for shortening pathogen identification, as they have shown high degrees of specificity and sensitivity, thus reducing mortality and the length of hospitalization and ICU stay of patients. However, these techniques are not sufficient to differentiate sepsis from other inflammatory processes, and there are no biomarkers able to identify only septic patients. 

Of importance, as also reported by Lazzaro et al., the two cornerstones of sepsis treatment are empiric antibiotic therapy and infection control. Numerous studies have shown that appropriate initial antibiotic therapy reduces mortality rates more than any other medical procedure, and many studies suggest that mortality rates progressively increase, considering a diagnostic window from 1 to 24 h from the symptom-based recognition of sepsis to the start of antimicrobial treatment [9]. Until the etiology of the infection is definitively assessed, a broad-spectrum therapy should be considered in order to cover both Gram-positive and Gram-negative bacteria. The right dosage of therapy is also a potential problem because most cases are observed in patients who are frail, in an intensive care unit, and are inotropic supported with multiple comorbidities, and it is very difficult to determine the PK/PD of the antimicrobials in these special populations. The rapid spread of MDR microorganisms, due mainly to the disproportionate use of antibiotics, highlights an urgent issue about the management and treatment of sepsis, especially in countries with scarce economic resources [10]. Ideal techniques should include a number of features, such as rapid microbiological results, low invasiveness, high sensitivities, and high specificities, but also the ability to differentiate between host- and pathogen-mediated inflammatory responses to influence clinical management to obtain a targeted therapeutic approach as soon as possible [11,12,13]. Due to the difficulty in understanding the mechanisms of sepsis and limitations in current diagnostic procedures, there is great interest in identifying promising biomarkers that can not only predict mortality rates but also be used as indices of the early detection of inflammation status. Moreover, as sepsis and septic shock are characterized by a dysfunction of the immune response, adjunctive immune-modulatory treatments have been developed in support of antibiotic therapies to restore immune response. Single adjunctive therapies have been studied and are currently being evaluated in clinical trials, with discordant results [8]. However, it should be emphasized that the now-available knowledge regarding management, diagnostic techniques, and precision medicine has not yet been fully elucidated, and current studies are sometimes discordant. Therefore, further studies will be needed to more clearly understand these aspects in order to implement more appropriate diagnostic and therapeutic strategies [14,15,16,17,18,19,20]. 

## 4. Conclusions

In conclusion, crucial aspects of sepsis were highlighted in this Special Issue, including the mechanisms underlying sepsis; the risk factors for the development of sepsis and septic shock; the advances in diagnoses, managements, and therapies of these severe infections; and the role of adjunctive therapies for the treatment of septic patients. A timely empirical antimicrobial treatment and the importance of definitive anti-infective therapy with in vitro activity against the microbial isolates are crucial to improving survival, emphasizing the importance of an adequate and the early source control of infection. Moreover, the pharmacokinetic and pharmacodynamic properties of antibiotics should be considered because of changes in clearance and volume of distribution that are frequently observed in critically ill patients, with the potential to influence the concentration of the drug at the site of infections.

## 5. Take-Home Messages

- As reported in the studies published in this Special Issue, the mechanisms of sepsis may be mainly based on the activation of a hyperinflammatory innate immune system response to microorganisms, including multidrug-resistant pathogens.
- As a consequence, microorganisms can directly and indirectly stimulate endothelial activation and humoral changes, including the involvement of the innate and the adaptive immune systems.
- Blood cultures remain the gold-standard diagnostic laboratory technique for the diagnosis of sepsis, but molecular techniques are essential to obtain early microbiological reports.
- Biomarkers are important tools to differentiate sepsis from noninfectious diseases and thereby contribute to early diagnosis.
- Prompt empirical broad-spectrum antibiotic therapy and source control of infection are the most effective treatment strategies in sepsis.
- Pharmacokinetic/pharmacodynamic adjustments are recommended for patients with specific characteristics, including neonates, obesity, burns, and altered renal functions.
